# Signaling Pathways Related to Protein Synthesis and Amino Acid Concentration in Pig Skeletal Muscles Depend on the Dietary Protein Level, Genotype and Developmental Stages

**DOI:** 10.1371/journal.pone.0138277

**Published:** 2015-09-22

**Authors:** Yingying Liu, Fengna Li, Xiangfeng Kong, Bie Tan, Yinghui Li, Yehui Duan, François Blachier, Chien-An A. Hu, Yulong Yin

**Affiliations:** 1 Key Laboratory of Agro-ecological Processes in Subtropical Region, Chinese Academy of Sciences, Changsha, Hunan, China; 2 Hunan Animal Science and Veterinary Medicine Research Institute, Changsha, China; 3 INRA, CNRH-IdF, AgroParisTech, UMR 914 Nutrition Physiology and Ingestive Behavior, Paris, France; 4 Department of Biochemistry and Molecular Biology, University of New Mexico, Albuquerque, United States of America; 5 University of Chinese Academy of Sciences, Beijing, China; 6 School of Biology, Hunan Normal Univesity, Hunan, Changsha City, 410018, China; 7 Changsha Lvye Biotechnology Limited Company, Guangdong Hinapharm Group and WangDa Academician Workstation, Hunan, Changsha City, 41019, P. R. China; Monash University, AUSTRALIA

## Abstract

Muscle growth is regulated by the homeostatic balance of the biosynthesis and degradation of muscle proteins. To elucidate the molecular interactions among diet, pig genotype, and physiological stage, we examined the effect of dietary protein concentration, pig genotype, and physiological stages on amino acid (AA) pools, protein deposition, and related signaling pathways in different types of skeletal muscles. The study used 48 Landrace pigs and 48 pure-bred Bama mini-pigs assigned to each of 2 dietary treatments: lower/GB (Chinese conventional diet)- or higher/NRC (National Research Council)-protein diet. Diets were fed from 5 weeks of age to respective market weights of each genotype. Samples of *biceps femoris* muscle (BFM, type I) and *longissimus dorsi* muscle (LDM, type II) were collected at nursery, growing, and finishing phases according to the physiological stage of each genotype, to determine the AA concentrations, mRNA levels for growth-related genes in muscles, and protein abundances of mechanistic target of rapamycin (mTOR) signaling pathway. Our data showed that the concentrations of most AAs in LDM and BFM of pigs increased (*P*<0.05) gradually with increasing age. Bama mini-pigs had generally higher (*P*<0.05) muscle concentrations of flavor-related AA, including Met, Phe, Tyr, Pro, and Ser, compared with Landrace pigs. The mRNA levels for myogenic determining factor, myogenin, myocyte-specific enhancer binding factor 2 A, and myostatin of Bama mini-pigs were higher (*P*<0.05) than those of Landrace pigs, while total and phosphorylated protein levels for protein kinase B, mTOR, and p70 ribosomal protein S6 kinases (p70S6K), and ratios of p-mTOR/mTOR, p-AKT/AKT, and p-p70S6K/p70S6K were lower (*P*<0.05). There was a significant pig genotype-dependent effect of dietary protein on the levels for mTOR and p70S6K. When compared with the higher protein-NRC diet, the lower protein-GB diet increased (*P*<0.05) the levels for mTOR and p70S6K in Bama mini-pigs, but repressed (*P*<0.05) the level for p70S6K in Landrace pigs. The higher protein-NRC diet increased ratio of p-mTOR/mTOR in Landrace pigs. These findings indicated that the dynamic consequences of AA profile and protein deposition in muscle tissues are the concerted effort of distinctive genotype, nutrient status, age, and muscle type. Our results provide valuable information for animal feeding strategy.

## Introduction

It is economically important to increase the rate and speed of skeletal muscle growth in animals raised for meat including pigs. The growth of animals is a net result of complicated metabolic and physiological network including synthesis and utilization of amino acids (AA) [1], intracellular protein turnover and deposition, as well as their regulation by nutrients, age, endocrine and exocrine secretion and other factors. The skeletal muscle, which accounts for 20–50% of total body mass among the different pig genotypes, is the major metabolic tissue, contributing up to 40% of the resting metabolic rate in adult pigs [2,3]. In addition, muscle cell lineage determination and differentiation require coordinated extracellular and intracellular signaling events that converge upon the nuclear genome to coordinate, depending on the intracellular content composition, specific patterns of gene expression required for normal cellular homeostasis [4]. Such programs of gene transcription require cell-specific and more widely expressed DNA binding transcription factors and their attending co-regulators that act on the epigenome for appropriate control of gene expression [5].

Dietary AAs are not only substrates for protein synthesis but also exert signaling effects on muscle protein deposition [6–13]. It is well known that cell signaling via the mechanistic target of rapamycin (mTOR; a highly conserved serine/threonine protein kinase) is a major mechanism for regulation of protein synthesis in cells [14]. The mTOR integrates extracellular signals, then phosphorylates the downstream targets, such as p70 ribosomal protein S6 kinases (p70S6K) and eukaryotic initiation factor 4E binding protein 1 (EIF-4EBP1). These coordinately affect gene transcription and protein translation, which are involved in the regulation of cell growth, proliferation and differentiation. Recently, mTOR was found to act as a sensor for cell growth regulated by AA [15,16]. Since chronic feeding of a low-protein diet could impair translation initiation activation and reduce protein synthesis through the mTOR signaling pathway in pigs [17], supplementation strategy with crystalline AA to low-protein diet was reported to be efficient for stimulating tissue protein synthesis via the mTOR pathway [8,18]. Thus, dietary protein concentration, especially level of protein or free AA, is very important as a modulator for the protein deposition and muscle growth.

Genetic background can influence growth and nutrient requirements in animals, including pigs. Bama mini-pig (*Sus scrofa domestica*), a Chinese indigenous mini-pig breed located in Bama County, Guangxi Province of China produces high-quality meat. Landrace is a fast-growing lean genotype with commercial traits. In our previous investigations [10], Landrace pigs showed faster growth rate and better muscle growth than Bama mini-pig. In contrast, Bama mini-pigs presented higher quality meat traits but more fat deposition capacity together with lower growth rate than Landrace pigs. Therefore, finding the optimal balance between economic aspect of pig production and meat nutritional quality is the goal of many researchers. Pig skeletal muscle fiber type varies with anatomical location. For example, *biceps femoris* muscle (BFM) and *longissimus dorsi* muscle (LDM) mainly contain type I and type II fiber, respectively [19]. We hypothesized that the difference between these two genotypes of pigs in their muscle growth, meat quality, and intermuscular adipose deposition [10] may lead to dietary protein-dependent differences in protein deposition and related signaling pathways in different types of skeletal muscles. The main objective of the current study was to evaluate the effects of genotype, diet, and age on protein deposition, and growth of different muscle fiber types as well as the associated regulating signaling pathways.

## Materials and Methods

### Animals, diets, and treatments

Ninety-six barrows [48 purebred Bama mini-pigs (fatty type; average initial body weight (BW), 3.38 ± 0.96 kg), and 48 Landrace pigs (lean type; average initial BW, 7.68 ± 0.89 kg)] were fed from 5 weeks of age up to market weight. The experiment was a 2 × 2 factorial arrangement, with 2 genotypes (Bama mini-pigs *vs*. Landrace pigs) and 2 dietary protein levels (NRC diet *vs*. lower protein-Chinese conventional diet [GB]), resulting 4 different treatments (Table 1). The piglets from each genotype were randomly assigned to one of the two dietary treatments, with 24 piglets in each treatment. The NRC diets were formulated to meet NRC [20] recommended nutrient requirements, whereas the lower protein-GB diets were formulated to meet recommendations of Chinese National Feeding Standard for Swine [21], and with a protein level of the latter being lower than the former (Table 2). The AA compositions in each diet which were determined according to our previous method [22] are shown in Table 3. All animals were individually housed in 0.6 m × 1.2 m pens with hard plastic slatted flooring [23]. Each pen was equipped with a stainless-steel feeder and a nipple drinker [24]. The room temperature was maintained at 25–27°C [25]. All pigs had *ad libitum* access to drinking water, and were fed three times daily (0800, 1300, and 1800). Dietary phase was based on the physiological stage of pigs [26].

**Table 1:** Experimental design.

Item	Landrace pig		Bama mini-pig	
	GB diet group	NRC diet group	GB diet group	NRC diet group
Nursery phase^1^	GB diet 1	NRC diet 1	GB diet 1	NRC diet 1
Growing phase^2^	GB diet 2	NRC diet 2	GB diet 2	NRC diet 2
Finishing phase^3^	GB diet 3	NRC diet 3	GB diet 3	NRC diet 3

^1, 2, 3^ Body weight ranges for nursery, growing, and finishing phases were defined as 7–20, 20–50, and 50–90 kg, respectively, for Landrace pigs, and 3–15, 15–35, and 35–55 kg, respectively, for Bama mini-pigs. GB diet, lower protein-Chinese conventional diet; NRC diet, higher protein-NRC diet.

**Table 2:** Ingredients and nutrient levels of experimental diets.

Item	NRC diet 1	NRC diet 2	NRC diet 3	GB diet 1	GB diet 2	GB diet 3
Ingredients (%)						
Corn	62.80	66.00	69.50	63.00	60.00	66.00
Soybean meal, 42% CP	26.00	28.00	23.00	25.00	26.50	21.00
Fish meal, 62% CP	7.00	2.00	-	3.00	-	-
Wheat bran	-	-	3.00	6.34	10.75	10.50
Soybean oil	1.95	1.50	2.10	-	-	-
CaHPO_4_	0.45	0.70	0.65	0.80	0.80	0.50
CaCO_3_	0.50	0.50	0.45	0.56	0.65	0.70
Salt	0.30	0.30	0.30	0.30	0.30	0.30
Premix*	1.00	1.00	1.00	1.00	1.00	1.00
Nutrient levels						
Digestible energy (MJ/kg)	14.22	14.21	14.22	13.46	13.40	13.40
Crude protein^†^ (%)	20.06	18.01	15.11	18.03	16.05	13.46
Calcium (%)	0.75	0.62	0.50	0.69	0.62	0.56
Available phosphorus (%)	0.39	0.28	0.21	0.21	0.13	0.12

*Premix provided for 1 kg of complete diet: Cu (as copper sulfate), 10 mg; Fe (as ferrous sulfate), 100 mg; Se (as sodium selenite), 0.30 mg; Zn (as zinc oxide), 100 mg; Mn (as manganese sulfate), 10 mg; V_D3_, 9.65 μg; V_A_, 925.8 μg; V_E_, 15.4 mg; V_K3_, 2.3 mg; V_B2_, 3.9 mg; D-calcium pantothenate, 15.4 mg; nicotinic acid, 23 mg; choline, 80 mg; V_B12_, 0.016 mg.

^†^Crude protein was determined value, other nutrients were calculated values.

**Table 3:** Analyzed AA composition of the experimental diets (mg/g, as-fed basis).

Item	NRC diet 1	NRC diet 2	NRC diet 3	GB diet 1	GB diet 2	GB diet 3
Essential AA						
Arg	9.65	9.25	7.53	8.66	8.79	6.86
His	4.97	4.83	3.94	4.66	4.45	3.66
Ile	5.68	5.47	4.48	5.17	5.19	3.97
Leu	14.95	15.07	12.78	14.57	13.67	12.11
Lys	8.56	8.03	6.12	7.61	7.94	5.50
Met	2.49	1.77	2.13	2.32	1.74	1.34
Phe	6.90	7.47	5.78	6.88	6.96	5.50
Thr	6.06	5.58	4.39	5.44	5.28	4.06
Val	8.20	7.20	6.51	7.66	6.77	5.66
Total EAA	67.46	64.68	53.66	62.98	60.78	48.63
Non-essential AA						
Ala	11.26	9.19	8.80	10.59	8.74	7.84
Asp*	16.43	16.16	12.96	15.40	15.39	12.06
Cys	3.42	2.54	3.21	3.41	2.42	2.66
Glu**	37.06	38.02	31.06	37.06	35.94	29.94
Gly	7.66	6.77	5.30	6.89	6.36	4.91
Pro	18.76	18.18	14.94	17.91	16.99	14.00
Ser	5.90	6.43	4.62	5.72	5.86	4.57
Tyr	5.29	5.19	4.49	4.67	4.77	4.06
Total NEAA	105.78	102.50	85.38	101.66	96.48	80.03
Total AA	173.23	167.18	139.05	164.63	157.26	128.66

*Including aspartate and asparagine;

**Including glutamate and glutamine.

The experiment was carried out in accordance with the Chinese guidelines for animal welfare and experimental protocols, and approved by the Animal Care and Use Committee of the Institute of Subtropical Agriculture, the Chinese Academy of Sciences.

### Sample collection

Body weight ranges for nursery, growing, and finishing phases were defined as 7–20, 20–50, and 50–90 kg, respectively, for Landrace pigs, and 3–15, 15–35, and 35–55 kg, respectively, for Bama mini-pigs (Table 1). At the end of each phase, 8 pigs from each treatment were randomly selected for blood collection, and euthanized [27]. Briefly, the pigs were held under general anesthesia and killed by injection of 4% sodium pentobarbital solution (40 mg/kg BW) into the jugular vein. After removing the head, legs, tail, and viscera, the carcass was split longitudinally. Samples of LDM and BFM on the right-side carcass were collected immediately, and the visible intermuscular adipose tissue was carefully removed. The samples were snap-frozen in liquid nitrogen, and stored at -80°C for further analysis [28].

### Determination of AA

Approximately 0.1 g freeze-dried muscle was ground and hydrolyzed in 10 ml of 6 mol/L hydrochloric acid solution at 110°C for 24 h. The solution was diluted with water to 100 ml and 1 ml of the supernatant was used for analysis [29,30]. The samples were filtered through a 0.45-μm membrane before analysis [31] by an ion-exchange AA analyzer (L8800, Hitachi, Tokyo, Japan).

### RNA extraction and cDNA synthesis

Total RNA was isolated from LDM and BFM tissues using the TRIzol reagent (Invitrogen-Life Technologies, Carlsbad, CA, USA) and treated with DNase I (Invitrogen) according to the manufacturer’s instructions. The RNA quality was checked by 1% agarose gel electrophoresis, and stained with 10 μg/ml ethidium bromide [32]. The RNA was shown to have an OD260:OD280 ratio between 1.8 and 2.0. The first-strand cDNA was synthesized with Oligo (dT) 20 and Superscript II reverse-transcriptase (Invitrogen), according to the manufacturers’ instructions.

### Analysis of muscle growth-related gene expression

Primers for myogenic determining factor (MyoD), myogenin (MyoG), myocyte-specific enhancer binding factor 2 A (MEF2A), and myostatin (MSTN) (Table 4) were designed using the Primer 5.0 software. Real-time reverse-transcription polymerase chain reaction (RT-PCR) was performed using the SYBR Green detection kit (TaKaRa, Japan), containing MgCl_2_, dNTP, and HotStar Taq Polymerase. An aliquot (2 μl) of a cDNA template (equal to 25 ng of total RNA) solution was added to a total volume of 10 μl containing 5 μl SYBR Green mix, 0.2 μl ROX Reference Dye (50 X), and 0.2 μl each of forward and reverse primers. After a pre-denaturation program (10 s at 95°C), 40 cycles of amplification were performed (95°C for 10 s, 60°C for 20 s), followed by a melting curve program (60–99°C with a heating rate of 0.1°C/s and fluorescence measurement), the fluorescent signal was detected by the ABI Prism 7900HT (Applied Biosystems, Marsiling Industrial Estate Road 3, Singapore). A melting curve was generated for each sample at the end of each run to ensure the purity of the amplified products. The amplification of glyceraldehyde-3-phosphate dehydrogenase (GAPDH) in each sample was used to normalize the mRNA levels for the target genes. The relative expression ratio (R) of mRNA was calculated by the following formula:

$$R\;=\;2^{–}{{}^{\Delta \Delta }}^{Ct\;\left(sample–control\right)},$$

where ΔΔC_t_ (sample—control) = (C_t_ target genes—C_t_ GAPDH) for the sample—(C_t_ target genes—C_t_ GAPDH) for the control.

**Table 4:** Primers used for real-time PCR.

Gene	Accession no.	Primers	Size (bp)
MyoD	NM_001002824	F: 5'-CAACAGCGGACGACTTCTATG-3'	383
		R: 5'-GCGCAAGATTTCCACCTT-3'	
MyoG	NM_001012406	F: 5'-AGGCTACGAGCGGACTGA-3'	230
		R: 5'-GCAGGGTGCTCCTCTTCA-3'	
MEF2A	NM_001099698	F: 5'-TGAATACCCAGAGGATAAGCAGTT-3'	133
		R: 5'-TAATCGGTGTTGTAGGCGG-3'	
MSTN	AY448008	F: 5'-GTCCCGTGGATCTGAATG-3'	293
		R: 5'-TTCCGTCGTAGCGTGATA-3'	
GAPDH	NM_001206359	S: 5'-AAGGAGTAAGAGCCCCTGGA-3'	140
		A: 5'-TCTGGGATGGAAACTGGAA-3'	

MyoD, myogenic differentiation factor; MyoG, myogenin; MEF2A, myocyte specific-enhancer factor-2A; MSTN, myostatin; GAPDH, glyceraldehyde-3-phosphate dehydrogenase.

Real-time RT-PCR efficiencies were determined by the amplification of a dilution series of cDNA according to the equation 10^(-1/slope)^, as described by Liu et al. [10], and were consistent between target genes and GAPDH. Negative controls were also used, in which cDNA solution was replaced by an equal volume of water.

### Analysis of mTOR-pathway proteins

The frozen muscle samples were powdered in liquid nitrogen, and lysed in RIPA buffer (150 mM NaCl, 1% Triton X-100, 0.5% sodium deoxycholate, 0.1% SDS, and 50 mM Tris-HCl at pH 7.4), containing a protease inhibitor cocktail purchased from Roche (Shanghai, China). After centrifugation at 10, 000 ×*g* and 4°C for 10 min, protein concentration in the supernatant fluid was determined using the Bicinchoninic Acid assay (Beyotime Biotechnology, Haimen, China). All samples were diluted to an equal protein concentration with 2 × loading buffer (0.63 ml of 0.5 M Tris-HCl (pH 6.8), 0.42 ml 75% glycerol, 0.125 g sodium dodecyl sulfate (SDS), 0.25 ml β-mercaptoethanol, 0.2 ml 0.05% solution of bromphenol blue, and 1 ml water to a final volume of 2.5 ml), and heated in boiling water for 5 min. After cooling on ice, the samples were used for Western blot analysis.

Same amounts of sample aliquots (20 μg protein) were subjected to SDS-PAGE (4%–12% gradient gel) and were then transferred to PVDF membranes (Millipore, MA, USA) overnight at 12 V using the Bio-Rad Transblot apparatus (CA, USA). The membranes were blocked in 5% fat-free milk in Tris-Tween buffered saline (TTBS; 20 mM Tris/150 mM NaCl, pH 7.5, and 0.1% Tween-20) for 3 h and then incubated with the primary antibodies (Table 5) at 4°C overnight with gentle rocking. After washing three times with TTBS, the membranes were incubated at room temperature for 2 h with horseradish peroxidase-linked secondary antibodies (Santa Cruz, CA, USA). The dilution of secondary antibodies was 1:5, 000. Finally, the membranes were washed with TTBS, followed by development using Super-signal West Dura Extended Duration Substrate according to the manufacturer’s instructions (Pierce, Rockford, IL). The images were detected on chemiluminescence (Applygen Technologies Inc., Beijing, China). Multiple exposures of each Western blot were performed to ensure linearity of chemiluminescence signals. Western blots were quantified by measuring the intensity of bands with correct molecular weight using AlphaImager 2200 software (Alpha Innotech Corporation, CA, USA). The ratio of intensities of a target protein band and housekeeping protein band was calculated for each filter and the ratios from the different Western blot filters were used for analyzing the relative abundances of target proteins.

**Table 5:** Antibodies and dilution used for Western blot analyses.

Antibody	Catalog number	Dilution
Rabbit polyclonal anti-AKT	CST#9272	1 : 1000
Rabbit polyclonal anti-phospho-AKT (Ser473)	CST#9271S	1 : 1000
Rabbit polyclonal anti-mTOR	CST#2972	1 : 1000
Rabbit monoclonal anti-phospho-mTOR (Ser2448)	CST#5536	1 : 1000
Rabbit polyclonal anti-p70S6K	SC-9027	1 : 400
Mouse monoclonal anti-phospho-p70S6K	SC-8416	1 : 400
Rabbit polyclonal anti-4EBP1	SC-6936	1 : 400
Rabbit polyclonal anti-phospho-4EBP1 (Thr70)	SC-18092-R	1 : 400
Mouse monoclonal anti-β-actin	SC-47778	1 : 1000
Horseradish-peroxidase-linked anti-rabbit IgG	sc-2027	1 : 5000
Horseradish-peroxidase-linked anti-mouse IgG	sc-2025	1 : 5000

### Statistical analysis

Data were analyzed by multifactor ANOVA using the GLM procedure of SAS 9.1 (SAS Institute Inc., Cary, NC) and means were separated using Tukey’s method. The effects of pig genotype, diet, physiological stage, and their interactions were taken into consideration. Differences between means were considered as statistically significant at *P*<0.05 and a trend toward significance at *P*<0.10.

## Results

### Muscle AA concentration

Muscle AA concentrations are shown in Tables 6 and 7. In general, the concentrations of most AA, total AA (TAA), essential AA (EAA), and flavor AA (FAA) in LDM and BFM increased in an age-dependent manner, regardless of pig genotype and dietary protein level. Pig genotype affected most indices of AA pool of different muscle tissues throughout the trial. Diet had significant effects on several AA.

**Table 6:** Effects of genotype, diet, and developmental phase on amino acid concentrations in *longissimus dorsi* muscle of pigs (mg/g, as-fresh basis; n = 8).

	Nursery				Growing				Finishing				*P*-value						
Item	Landrace		Bama mini-pig		Landrace		Bama mini-pig		Landrace		Bama mini-pig		*P*_P_	*P*_G_	*P*_P*G_	*P*_D_	*P*_P*D_	*P*_G*D_	*P*_P*G*D_
	GB diet	NRC diet	GB diet	NRC diet	GB diet	NRC diet	GB diet	NRC diet	GB diet	NRC diet	GB diet	NRC diet							
Asp	17.12±0.42	16.52±0.72	17.82±0.74	17.68±0.52	19.53±0.40	19.74±0.39	17.58±1.32	11.14±0.77	21.10±0.34	21.04±0.67	14.63±3.18	11.50±0.31	0.10	**<.01**	**<.01**	**<.01**	0.40	0.13	**<.01**
Thr	8.76±0.21	8.86±0.19	9.06±0.36	9.02±0.28	10.19±0.28	9.67±0.29	9.27±0.49	8.88±0.89	10.64±0.14	10.72±0.38	10.85±0.10	10.98±0.25	**<.01**	0.54	**0.05**	0.62	0.50	0.98	0.96
Ser	6.83±0.19	6.49±0.25	7.38±0.30	7.21±0.19	7.16±0.26	6.46±0.31	8.19±0.48	8.02±0.25	8.28±0.12	8.43±0.30	8.56±0.10	8.49±0.17	**<.01**	**<.01**	**0.04**	0.21	0.55	0.62	0.70
Glu	34.48±0.78	35.27±1.29	35.12±1.35	34.67±0.91	40.32±0.82	40.59±0.93	39.98±1.86	36.93±2.18	40.42±0.70	40.13±1.22	40.86±0.36	40.12±1.37	**<.01**	0.42	0.37	0.42	0.63	0.25	0.71
Gly	8.95±0.13	9.08±0.64	8.77±0.41	9.07±0.41	8.94±0.16	8.58±0.08	9.72±0.65	8.92±0.45	9.88±0.15	9.84±0.42	10.48±0.20	10.72±0.22	**<.01**	0.07	0.24	0.71	0.26	1.00	0.77
Ala	14.20±0.60	13.07±0.97	13.02±0.58	13.44±0.55	10.84±0.39	11.61±0.26	14.84±1.01	12.10±0.83	21.50±2.62	31.55±9.47	44.46±15.80	30.14±5.06	**<.01**	0.06	0.13	0.61	0.95	0.05	0.06
Val	8.70±0.24	9.30±0.42	8.00±0.37	8.31±0.42	9.39±0.47	10.63±0.37	9.92±0.58	8.94±0.57	10.36±0.22	10.46±0.33	11.02±0.33	10.75±0.20	**<.01**	0.21	0.10	0.51	0.67	0.06	0.19
Met	2.98±0.20	2.99±0.24	6.83±0.59	5.82±0.59	3.74±0.47	3.67±0.12	3.83±0.15	3.93±0.60	5.67±0.53	6.55±0.67	5.20±1.02	6.87±0.70	**<.01**	**<.01**	**<.01**	0.42	0.09	0.98	0.48
Ile	6.64±0.35	7.42±0.63	7.42±0.27	7.18±0.24	8.98±0.25	9.35±0.19	7.54±0.46	7.23±0.47	8.25±0.28	8.47±0.21	8.65±0.13	8.47±0.17	**<.01**	**0.04**	**<.01**	0.60	0.83	0.09	0.83
Leu	13.57±0.56	14.09±0.72	16.20±0.66	15.02±0.53	16.17±0.37	16.60±0.32	14.72±1.13	13.58±1.04	16.75±0.69	16.97±0.62	17.33±0.15	17.27±0.37	**<.01**	0.99	**<.01**	0.62	0.90	0.15	0.75
Tyr	3.74±0.44	2.98±0.13	5.80±0.53	4.49±0.52	3.65±0.25	3.44±0.18	4.32±0.42	4.31±0.55	4.56±0.52	4.46±0.36	4.58±0.26	4.31±0.46	0.30	**<.01**	**0.03**	0.11	0.28	0.75	0.84
Phe	6.16±0.13	6.28±0.18	7.20±0.32	6.90±0.18	6.81±0.12	7.09±0.15	7.34±0.40	6.51±0.34	7.66±0.17	7.97±0.40	8.25±0.20	8.06±0.34	**<.01**	**0.01**	**0.05**	0.51	0.70	**0.03**	0.58
Lys	15.29±0.39	15.78±0.40	15.97±0.66	15.86±0.50	17.50±0.38	17.72±0.33	17.37±1.65	15.51±1.59	18.61±0.25	18.63±0.58	18.85±0.29	1900±0.20	**<.01**	0.69	0.21	0.66	0.52	0.30	0.55
His	8.35±0.21	8.08±0.21	7.59±0.39	7.63±0.33	9.67±0.17	9.51±0.21	10.23±0.82	9.11±1.02	10.29±0.30	9.93±0.38	9.92±0.13	10.23±0.10	**<.01**	0.44	0.41	0.29	0.54	0.98	0.36
Arg	11.45±0.22	11.65±0.22	11.60±0.45	11.55±0.33	12.72±0.30	12.61±0.20	11.21±2.33	12.21±0.63	13.36±0.15	13.33±0.47	13.57±0.15	13.85±0.19	**<.01**	0.64	0.38	0.59	0.91	0.62	0.75
Pro	10.58±1.14	13.00±1.50	44.95±4.96	37.50±4.57	11.02±1.77	13.41±1.61	13.68±2.10	18.25±1.74	24.76±3.94	33.55±3.62	34.40±1.33	36.46±1.07	**<.01**	**<.01**	**<.01**	0.32	0.26	0.26	0.45
TAA	177.79±3.88	175.85±4.08	222.71±10.07	211.36±5.33	196.62±4.77	200.67±2.88	199.75±8.90	185.55±6.51	232.09±8.69	252.01±14.39	261.61±14.94	247.23±10.08	**<.01**	**<.01**	**<.01**	0.55	0.74	**0.04**	0.61
EAA	81.90±2.03	84.44±3.00	89.85±3.51	87.28±2.31	95.16±2.26	96.84±1.67	91.44±6.87	85.88±5.45	101.59±1.72	103.01±3.60	103.64±0.59	105.48±2.38	**<.01**	0.96	**0.02**	0.96	0.77	0.31	0.74
NEAA	95.89±1.85	91.41±1.31	132.85±6.93	124.08±4.16	101.46±2.73	103.83±1.53	108.31±3.12	99.66±1.99	130.50±7.14	149.00±12.01	157.97±14.66	141.75±7.72	**<.01**	**<.01**	**<.01**	0.44	0.70	**0.03**	0.25
FAA	86.20±1.48	80.59±1.17	86.33±3.45	86.42±2.52	92.34±1.91	93.13±1.66	93.34±4.76	81.30±3.69	106.25±3.10	115.89±10.58	124.00±15.12	106.34±6.62	**<.01**	0.84	0.32	0.15	0.90	**0.04**	0.06
EAA/TAA	0.46±0.002	0.48±0.006	0.40±0.007	0.42±0.009	0.48±0.004	0.48±0.004	0.46±0.017	0.46±0.014	0.44±0.010	0.41±0.015	0.40±0.023	0.43±0.009	**<.01**	**<.01**	**<.01**	0.32	0.42	0.12	0.09
EAA/NEAA	0.85±0.006	0.92±0.025	0.68±0.022	0.71±0.026	0.94±0.014	0.93±0.014	0.84±0.055	0.86±0.050	0.79±0.034	0.71±0.043	0.67±0.061	0.75±0.024	**<.01**	**<.01**	**<.01**	0.35	0.46	0.23	0.09

P, phase; G, genotype; P × G, phase ×genotype interaction; D, diet; P × D, phase × diet interaction; G × D, genotype × diet interaction; P × G × D, phase ×genotype × diet interaction; TAA, total AA; EAA, essential AA, including Arg, His, Ile, Met, Lys, Val, Leu, Phe, and Thr; NEAA, non-essential AA, including Asp, Ser, Glu, Gly, Ala, Tyr, and Pro; FAA, flavor AA, including Asp, Glu, Gly, Ala, and Arg.

Data were means plus pooled SEM. Effects were considered statistically significant when *P*<0.05.

**Table 7:** Effects of genotype, diet, and developmental phase on amino acid concentrations in *biceps femoris* muscle of pigs (mg/g, as-fresh basis; n = 8).

	Nursery				Growing				Finishing				*P*-value						
Item	Landrace		Bama mini-pig		Landrace		Bama mini-pig		Landrace		Bama mini-pig		*P*_P_	*P*_G_	*P*_P*G_	*P*_D_	*P*_P*D_	*P*_G*D_	*P*_P*G*D_
	GB diet	NRC diet	GB diet	NRC diet	GB diet	NRC diet	GB diet	NRC diet	GB diet	NRC diet	GB diet	NRC diet							
Asp	16.13±0.55	15.59±0.25	16.98±0.55	17.51±0.49	18.58±0.23	17.15±1.17	18.70±1.55	17.79±0.41	20.17±0.43	20.80±0.33	16.95±1.95	21.64±1.08	**<.01**	0.66	0.06	0.26	**<.01**	**0.03**	0.23
Thr	8.08±0.21	8.06±0.10	8.58±0.24	8.86±0.25	9.63±0.22	8.88±0.47	10.65±0.71	9.15±0.22	10.31±0.20	10.78±0.12	10.14±0.35	11.13±0.47	**<.01**	**0.01**	0.37	0.62	**<.01**	0.93	0.33
Ser	5.97±0.19	6.22±0.13	7.06±0.23	7.18±0.17	6.72±0.29	6.11±0.27	8.59±0.64	7.21±0.14	7.99±0.17	8.29±0.10	7.74±0.30	8.74±0.45	**<.01**	**<.01**	**<.01**	0.74	**<.01**	0.83	0.19
Glu	33.65±1.54	31.87±0.66	33.83±1.11	34.12±0.92	38.10±0.78	35.91±2.36	41.34±3.09	34.50±0.75	38.90±0.73	40.42±0.49	36.86±0.98	41.52±2.07	**<.01**	0.50	0.67	0.38	**<.01**	0.91	0.13
Gly	8.20±0.23	7.93±0.09	8.62±0.19	8.67±0.18	8.27±0.14	7.79±0.50	10.01±0.72	8.74±0.22	9.48±0.24	9.92±0.12	9.57±0.34	10.46±0.35	**<.01**	**<.01**	**0.05**	0.53	**<.01**	0.98	0.27
Ala	12.28±0.53	12.96±0.53	13.61±0.64	14.29±0.91	10.73±0.49	10.10±0.89	17.87±1.42	13.03±0.75	20.32±1.84	32.39±3.69	28.93±5.25	25.14±3.05	**<.01**	**0.03**	0.22	0.51	**0.05**	**<.01**	**<.01**
Val	8.54±0.41	7.92±0.24	7.75±0.14	8.09±0.38	9.21±0.53	8.78±0.89	9.62±0.37	8.58±0.21	9.80±0.24	10.17±0.22	10.00±0.11	10.29±0.32	**<.01**	0.94	0.64	0.44	0.20	0.84	0.34
Met	2.66±0.12	2.61±0.08	5.32±0.66	5.76±0.25	3.71±0.44	3.54±0.52	3.72±0.39	2.95±0.09	5.95±0.77	6.56±0.52	5.63±0.47	5.00±0.42	**<.01**	**0.04**	**<.01**	0.72	0.57	0.39	0.39
Ile	7.12±0.48	6.42±0.32	7.26±0.30	7.45±0.22	8.23±0.20	8.18±0.61	8.50±0.94	6.89±0.23	8.11±0.33	8.85±0.15	8.24±0.46	8.86±0.47	**<.01**	0.84	0.17	0.57	0.05	0.58	0.10
Leu	14.34±0.60	13.91±0.35	16.50±0.85	16.56±0.59	15.25±0.25	15.67±1.03	21.10±3.38	15.31±0.59	17.07±0.90	19.05±0.68	17.65±1.68	20.77±2.07	**<.01**	**<.01**	0.63	0.88	**0.01**	0.27	0.06
Tyr	4.60±0.32	5.18±0.07	6.30±0.45	5.98±0.56	3.35±0.17	5.26±0.29	6.59±0.66	5.82±0.53	5.17±0.79	6.62±0.89	5.87±1.33	4.84±0.33	0.65	**<.01**	**0.01**	0.35	0.84	**<.01**	0.44
Phe	6.11±0.23	5.97±0.12	7.22±0.35	7.17±0.20	6.59±0.08	6.44±0.40	8.14±0.83	6.61±0.17	7.53±0.24	8.17±0.13	7.75±0.54	8.52±0.45	**<.01**	**<.01**	0.16	0.68	**<.01**	0.30	0.18
Lys	14.54±0.46	14.16±0.23	15.17±0.44	15.67±0.46	16.57±0.25	15.67±1.05	18.14±1.09	15.91±0.39	17.93±0.29	18.47±0.26	17.67±0.51	19.21±0.78	**<.01**	**0.02**	0.54	0.62	**<.01**	0.77	0.26
His	6.67±0.21	6.39±0.15	6.71±0.22	7.14±0.39	8.82±0.22	7.27±0.62	9.21±0.49	8.57±0.22	8.99±0.17	9.17±0.19	8.61±0.19	9.94±0.49	**<.01**	**0.01**	0.38	0.64	**<.01**	**0.02**	0.89
Arg	10.78±0.33	10.45±0.15	11.10±0.32	11.39±0.27	11.86±0.25	11.33±0.75	13.23±0.87	11.58±0.27	12.84±0.19	13.27±0.14	12.63±0.46	13.82±0.54	**<.01**	**0.02**	0.53	0.67	**<.01**	0.85	0.19
Pro	11.69±1.43	9.68±1.08	41.15±5.34	38.48±4.33	11.83±1.76	10.77±2.29	10.54±0.76	10.45±0.58	28.76±4.42	35.81±0.52	30.73±3.99	21.51±4.91	**<.01**	**<.01**	**<.01**	0.50	0.92	0.18	0.16
TAA	171.38±6.03	165.31±2.85	213.17±9.77	214.33±5.23	187.45±3.59	178.85±12.52	215.94±16.04	183.08±4.52	229.32±9.31	258.74±5.68	234.96±15.57	241.40±10.08	**<.01**	**<.01**	**<.01**	0.72	**0.01**	0.18	0.31
EAA	78.86±2.90	75.88±1.49	85.61±2.96	88.09±2.21	89.88±1.57	85.76±5.91	102.31±8.93	85.55±2.14	98.52±2.68	104.49±1.42	98.32±4.60	107.54±5.28	**<.01**	**<.01**	0.30	0.63	**<.01**	0.76	0.17
NEAA	92.52±3.19	89.44±1.42	127.56±6.98	126.24±4.20	97.57±2.15	93.09±6.74	113.63±7.13	97.54±2.42	130.80±6.72	154.25±4.57	136.64±11.05	133.86±7.37	**<.01**	**<.01**	**<.01**	0.82	**0.04**	0.06	0.19
FAA	81.04±2.40	78.80±0.79	84.14±1.91	85.99±2.29	87.53±1.22	82.28±5.48	101.14±6.40	85.64±1.89	101.72±2.61	116.80±3.24	104.94±5.93	112.59±4.68	**<.01**	**0.02**	0.18	0.89	**<.01**	0.23	0.24
EAA/TAA	0.46±0.003	0.46±0.002	0.40±0.008	0.41±0.009	0.48±0.003	0.48±0.005	0.47±0.006	0.47±0.002	0.43±0.008	0.40±0.006	0.42±0.009	0.45±0.015	**<.01**	**<.01**	**<.01**	0.91	0.78	**0.03**	**0.02**
EAA/NEAA	0.85±0.009	0.85±0.006	0.68±0.020	0.70±0.027	0.92±0.011	0.92±0.018	0.89±0.022	0.88±0.006	0.76±0.023	0.68±0.017	0.73±0.028	0.81±0.049	**<.01**	**<.01**	**<.01**	0.87	0.86	**0.03**	**0.02**

P, phase; G, genotype; P × G, phase ×genotype interaction; D, diet; P × D, phase × diet interaction; G × D, genotype × diet interaction; P × G × D, phase ×genotype × diet interaction; TAA, total AA; EAA, essential AA, including Arg, His, Ile, Met, Lys, Val, Leu, Phe, and Thr; NEAA, non-essential AA, including Asp, Ser, Glu, Gly, Ala, Tyr, and Pro; FAA, flavor AA, including Asp, Glu, Gly, Ala, and Arg.

Data were means plus pooled SEM. Effects were considered statistically significant when *P*<0.05.

As shown in Table 6, the concentrations of most AA (except of Asp and Tyr) in the LDM of both genotypes of pigs increased (*P*<0.05) with age. The concentrations of nonessential AA (NEAA) decreased (*P*<0.05) in growing phase but increased (*P*<0.05) in finishing phase. Ratios of EAA/TAA and EAA/NEAA increased (*P*<0.05) in growing phase but decreased (*P*<0.05) in finishing phase. The AA of LDM in Landrace pigs and Bama mini-pigs differed (*P*<0.05), including Asp, Ser, Met, Ile, Tyr, Phe, Pro, TAA, NEAA, and ratios of EAA/TAA and EAA/NEAA. Bama mini-pigs had higher (*P*<0.05) concentrations of Ser, Met, Ile, Tyr, Phe, and Pro during nursery phase, higher (*P*<0.05) concentrations of Ser, Met, Tyr, and Pro during growing phase, and higher (*P*<0.05) concentrations of Ile, Phe, and Pro during finishing phase; while lower (*P*<0.05) concentration of Asp was recorded during growing and finishing phases, when compared with Landrace pigs. Furthermore, the concentration of Asp in Bama mini-pigs fed the GB diet was higher (*P*<0.05) than those fed NRC diet, especially in growing and finishing phases. There were interactions between developmental phases and genotypes notably for Asp, Ser, Met, Ile, Leu, and Tyr. No interaction between developmental phases and diets was observed for any AA. An interaction between genotype and diet was evidenced for concentrations of Phe, TAA, NEAA, and FAA. In addition, according to the pig genotype, different responses were observed regarding interactions between developmental phases and diets for Asp level.

The BFM concentrations of most AA (except of Tyr) in Landrace pigs and Bama mini-pigs increased (*P*<0.05) with increasing age, as well as TAA, EAA, NEAA, and FAA of LDM (Table 7). There were marked differences in the concentrations of almost all AA between Landrace pigs and Bama mini-pigs, except for Asp, Glu Val, and Ile. When compared with Landrace pigs, Bama mini-pigs had higher concentrations of Thr, Ser, Gly, Ala, Leu, Phe, Lys, His, and Arg throughout the experimental period, as well as Met, Tyr, and Pro in the nursery phase, while lower concentrations of Met and Pro were measured in the finishing phase. Diet did not affect AA pool but there was interaction between pig genotype and developmental age on concentrations of Ser, Gly, Met, Tyr, Pro, TAA, NEAA, and ratios of EAA/TAA and EAA/NEAA. Interactions between the developmental phases and the diets were noted notably for Asp, Thr, Ser, Glu, Gly, and Ala. Interactions between genotypes and diets for Asp, Ala, Tyr, His, EAA/TAA and EAA/NEAA ratios, as well as an interaction between the developmental phases, genotypes, and diets was measured for the Ala concentration.

### Muscle growth-related genes expression

The mRNA levels for MyoD, MyoG, and MEF2A in LDM, and MEF2A in BFM increased (*P*<0.05) along the pig growth (Table 8). The mRNA levels for MyoD, MyoG, and MEF2A in LDM of Bama mini-pigs were much higher (*P*<0.05) than those in Landrace pigs. In addition, pigs fed the higher protein-NRC diet had higher (*P*<0.05) mRNA level for MyoG in BFM than those fed the lower protein-GB diet. There was interaction between pig genotype and developmental age on the mRNA level for MSTN in LDM, and MyoG and MEF2A in BFM.

**Table 8:** Effects of genotype, diet, and phase on expression of muscle growth-related genes in muscle tissues of pigs (n = 8).

	Nursery				Growing				Finishing				*P*-value						
Item	Landrace		Bama mini-pig		Landrace		Bama mini-pig		Landrace		Bama mini-pig		*P*_P_	*P*_G_	*P*_P*G_	*P*_D_	*P*_P*D_	*P*_G*D_	*P*_P*G*D_
	GB diet	NRC diet	GB diet	NRC diet	GB diet	NRC diet	GB diet	NRC diet	GB diet	NRC diet	GB diet	NRC diet							
*Longissimus dorsi* muscle																			
MyoD	0.55±0.12	0.83±0.11	0.87±0.23	2.12±0.33	1.37±0.16	2.56±0.43	2.99±0.25	1.31±0.19	3.28±0.18	2.52±0.15	3.33±0.21	2.94±0.30	**<.01**	**<.01**	0.13	0.91	**<.01**	0.09	**<.01**
MyoG	1.35±0.24	0.58±0.12	1.65±0.33	1.96±0.22	0.47±0.09	0.86±0.10	0.93±0.15	1.01±0.19	1.56±0.13	1.65±0.22	2.79±0.78	1.99±0.22	**<.01**	**<.01**	0.18	0.38	0.18	0.87	**<.01**
MEF2A	0.87±0.15	0.56±0.06	0.90±0.12	1.83±0.31	0.46±0.05	0.88±0.12	1.75±0.31	1.30±0.23	1.72±0.25	1.41±0.22	3.41±0.52	1.87±0.36	**<.01**	**<.01**	0.46	0.13	**<.01**	0.30	**<.01**
MSTN	0.83±0.13	0.44±0.08	1.10±0.17	2.39±0.28	0.66±0.08	0.62±0.14	1.36±0.19	1.43±0.17	1.87±0.24	1.99±0.29	2.98±0.55	1.38±0.24	**<.01**	**<.01**	**0.02**	0.47	**<.01**	0.93	**<.01**
*Biceps femoris* muscle																			
MyoD	2.12±0.31	1.89±0.23	2.41±0.40	2.25±0.11	1.79±0.44	2.13±0.63	1.41±0.37	1.36±0.36	0.94±0.21	3.41±0.83	1.74±0.46	1.56±0.40	0.23	0.28	0.22	0.13	0.07	**0.04**	0.06
MyoG	0.56±0.06	0.92±0.19	1.87±0.32	1.71±0.18	0.95±0.14	2.09±0.37	0.74±0.08	1.16±0.18	1.03±0.33	1.43±0.32	0.75±0.16	1.70±0.49	0.98	0.30	**<.01**	**<.01**	0.14	0.46	0.22
MEF2A	1.07±0.22	1.27±0.29	0.77±0.14	0.89±0.11	0.85±0.07	0.82±0.17	2.03±0.39	1.58±0.28	1.98±0.32	1.64±0.49	2.20±0.62	1.30±0.17	**<.01**	0.25	**<.01**	0.16	0.16	0.29	0.82
MSTN	0.91±0.21	1.48±0.56	2.13±0.42	1.95±0.41	1.14±0.14	1.63±0.31	2.68±0.60	2.21±0.23	1.24±0.28	1.09±0.24	1.26±0.35	0.90±0.25	**0.01**	**<.01**	0.08	0.93	0.67	0.13	0.76

P, phase; G, genotype; P × G, phase ×genotype interaction; D, diet; P × D, phase × diet interaction; G × D, genotype × diet interaction; P × G × D, phase ×genotype × diet interaction; MyoD, myogenic determining factor; MyoG, myogenin; MEF2A, myocyte-specific enhancer binding factor 2 A; MSTN, myostatin.

Data were means plus pooled SEM. Effects were considered statistically significant when *P*<0.05.

### Abundance of mTOR pathway proteins

**Fig 1:**
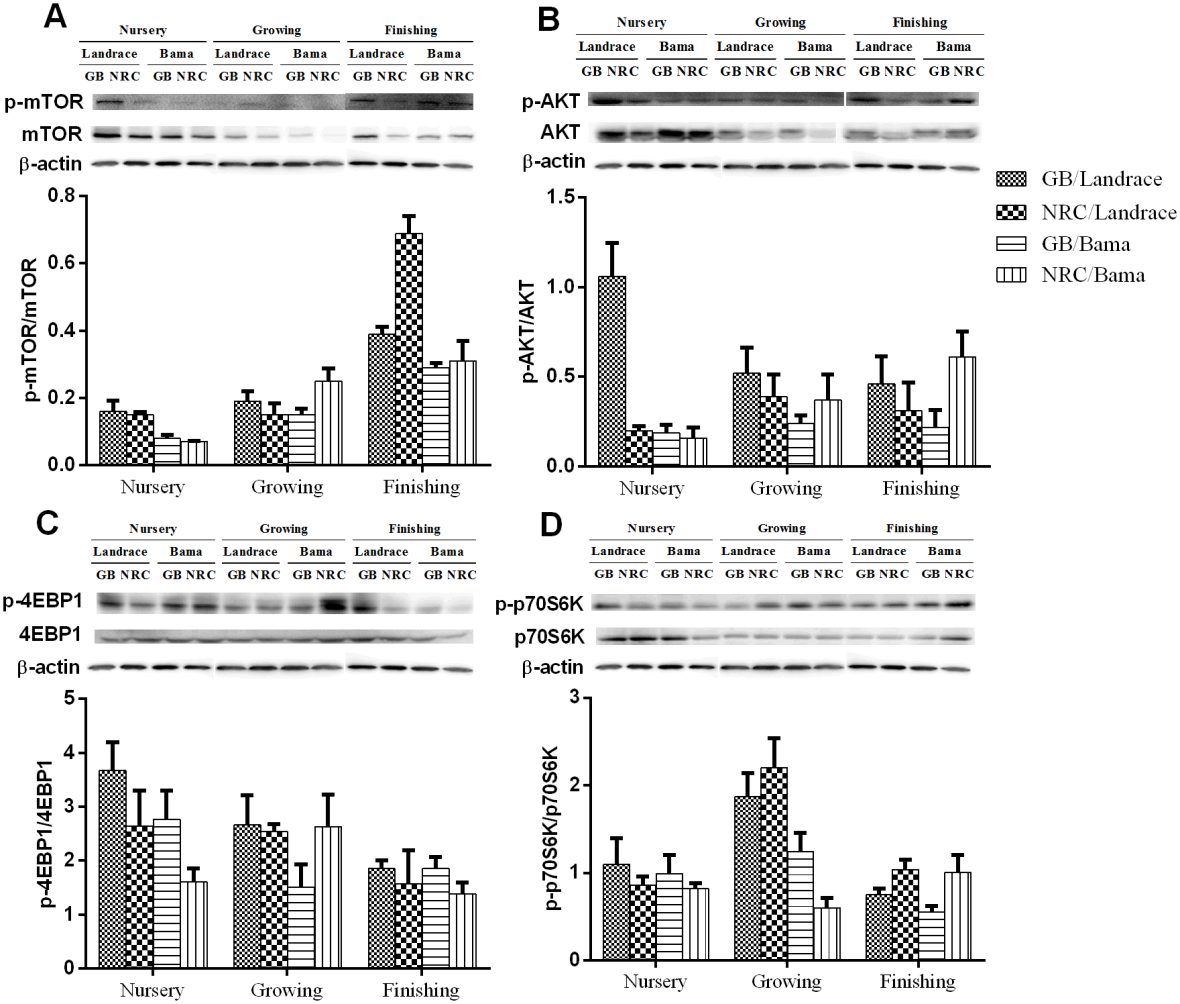
Representative Western blot analysis of total and phosphorylated (A) mTOR (Ser2448), (B) AKT (Ser473), (C) 4EBP1 (Thr70), (D) p70S6K, and β-actin in pig *longissimus dorsi muscle*. GB diet, lower protein-Chinese conventional diet; NRC diet, higher protein-NRC diet.

As shown in Fig 1 and Table 9, the protein abundances of mTOR, p-mTOR, protein kinase B (AKT), and p-AKT in the LDM decreased (*P*<0.05) in the growing phase and then increased in finishing phase. The protein abundances of 4EBP1 and p-4EBP1 decreased, while ratio of p-mTOR/mTOR increased (*P*<0.05) gradually along the pig development. In addition, according to the pig genotypes, different responses were observed regarding the abundances of the mTOR pathway proteins. The protein abundances of mTOR and p-mTOR in Landrace pigs were greater (*P*<0.05) than those of Bama mini-pigs, especially in the nursery and growing phases. The protein abundance of p-AKT in the nursery phase of Landrace pigs was greater (*P*<0.05) than that of Bama mini-pigs, while that of p70S6K in the finishing phase was lower (*P*<0.05). The ratios of p-mTOR/mTOR and p-AKT/AKT in nursery and finishing phases of Landrace pigs were greater than Bama mini-pigs, as well as p-p70S6K/p70S6K in growing and finishing phases. The lower protein-GB diet increased protein abundances of mTOR and p-mTOR throughout the whole trial, and of AKT in the nursery phase, when compared with the higher-NRC diet. The protein abundance of p70S6K in Landrace pigs fed the greater protein-NRC diet was higher (*P*<0.05) than in those fed the lower protein-GB diet. In contrast, the protein abundance of p70S6K in Bama mini-pigs fed the GB diet was higher (*P*<0.05) than those fed the NRC diet.

**Table 9:** *P*-values of ratios of phosphorylated- to total-protein abundance of mTOR pathway in *longissimus dorsi* muscle of pigs.

Item	*P*_P_	*P*_G_	*P*_P*G_	*P*_D_	*P*_P*D_	*P*_G*D_	*P*_P*G*D_
p-mTOR/mTOR^1^	**<.01**	**<.01**	**<.01**	**<.01**	**<.01**	0.24	**<.01**
p-AKT/AKT^2^	0.97	**0.04**	0.10	0.24	**0.04**	**<.01**	0.43
p-4EBP1/4EBP1^3^	0.07	0.13	0.59	0.36	0.18	0.66	0.63
p-p70S6K/p70S6K^4^	**<.01**	**<.01**	**<.01**	0.97	0.17	0.38	0.18

^1^ Ratio of phosphorylated- to total-protein abundance of mechanistic target of rapamycin;

^2^ ratio of phosphorylated- to total-protein abundance of protein kinase B;

^3^ ratio of phosphorylated- to total-protein abundance of 4E binding protein 1;

^4^ ratio of phosphorylated- to total-protein abundance of p70 ribosomal protein S6 kinase.

P, phase; G, genotype; P × G, phase ×genotype interaction; D, diet; P × D, phase × diet interaction; G × D, genotype × diet interaction; P × G × D, phase ×genotype × diet interaction.

**Fig 2:**
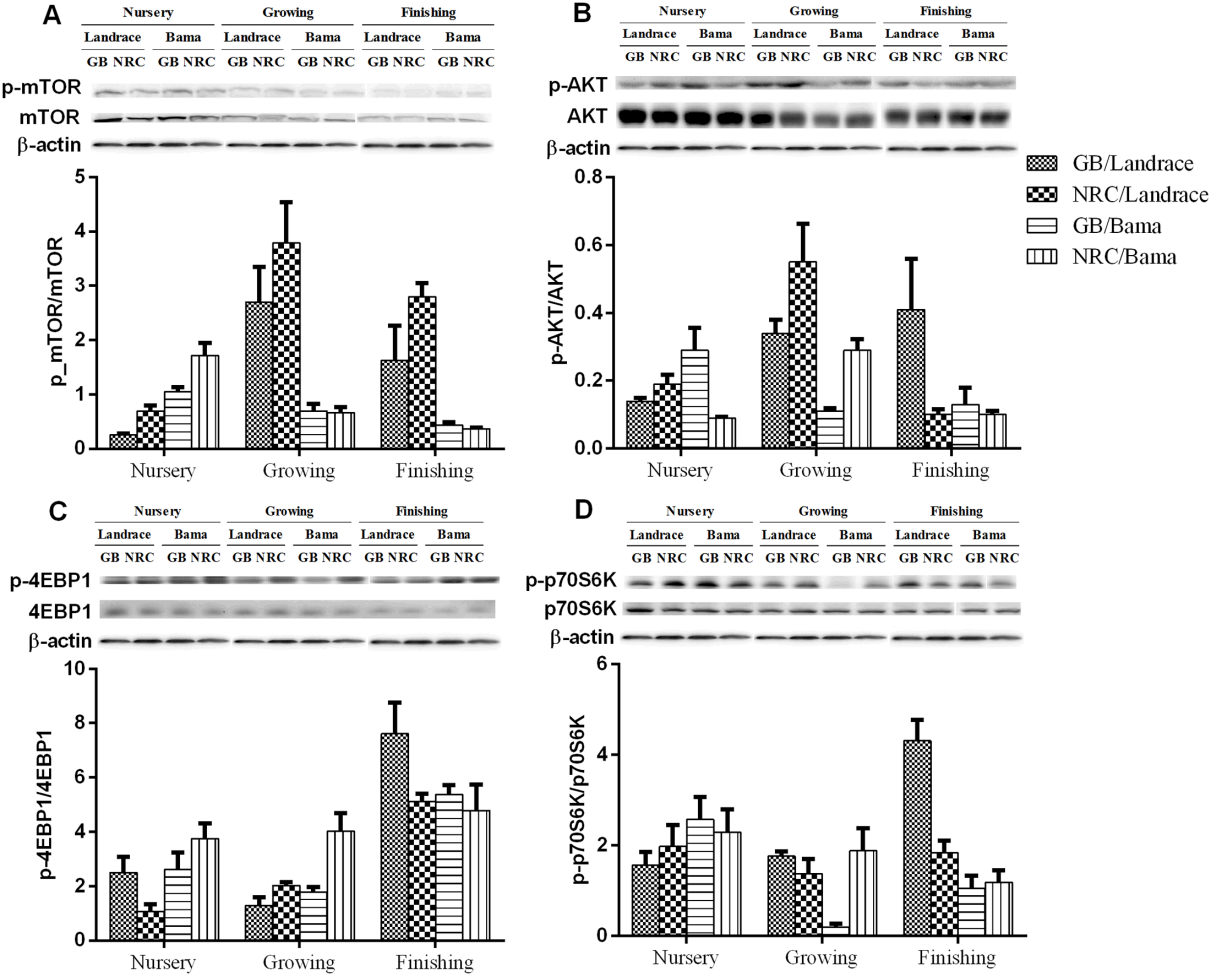
Representative Western blot analysis of the expression of the proteins listed in Fig 1 in pig *biceps femoris muscle*.

In the BFM, protein abundances of mTOR, p-mTOR, AKT, p-AKT, 4EBP1, p70S6K, and p-p70S6K decreased (*P*<0.05) with increasing age (Fig 2 and Table 10). The protein abundance of p-4EBP1, ratios of p-4EBP1/4EBP1 and p-p70S6K/p70S6K decreased in the growing phase, and increased in the finishing phase. Ratios of p-mTOR/mTOR and p-AKT/AKT increased in growing phase, and decreased in finishing phase (*P*<0.05). The protein abundances of AKT in the nursery and growing phases, and p-AKT in growing phase, were higher in Landrace than in Bama mini-pigs (*P*<0.05), while that of AKT and p-4EBP1 were lower in Landrace than in Bama mini-pigs (*P*<0.05) in the finishing phase. Ratios of p-mTOR/mTOR, p-AKT/AKT, and p-p70S6K/p70S6K of Landrace pigs were greater than those of Bama mini-pigs in the growing and finishing phases. The lower protein-GB diet increased (*P*<0.05) the protein abundance of mTOR in Bama mini-pigs, notably in the nursery and growing phases. The greater protein-NRC diet increased (*P*<0.05) the ratio of p-mTOR/mTOR of Landrace pigs, when compared with the GB diet.

**Table 10:** *P*-values of ratios of phosphorylated- to total-protein abundance of mTOR pathway in *biceps femoris* muscle of pigs.

Item	*P*_P_	*P*_G_	*P*_P*G_	*P*_D_	*P*_P*D_	*P*_G*D_	*P*_P*G*D_
p-mTOR/mTOR^1^	**<.01**	**<.01**	**<.01**	**0.01**	0.96	0.10	0.29
p-AKT/AKT^2^	**<.01**	**<.01**	**0.02**	0.61	**<.01**	0.98	**0.02**
p-4EBP1/4EBP1^3^	**<.01**	0.19	**<.01**	0.85	**<.01**	**<.01**	0.81
p-p70S6K/p70S6K^4^	**0.04**	**0.04**	**<.01**	0.59	**0.04**	**0.02**	**0.05**

^1^ Ratio of phosphorylated- to total-protein abundance of mechanistic target of rapamycin;

^2^ ratio of phosphorylated- to total-protein abundance of protein kinase B;

^3^ ratio of phosphorylated- to total-protein abundance of 4E binding protein 1;

^4^ ratio of phosphorylated- to total-protein abundance of p70 ribosomal protein S6 kinase.

P, phase; G, genotype; P × G, phase ×genotype interaction; D, diet; P × D, phase × diet interaction; G × D, genotype × diet interaction; P × G × D, phase ×genotype × diet interaction.

## Discussion

Due to its large mass (representing 40–45% of body weight), skeletal muscle is the largest reservoir of both peptide-bound and free AA in the body [33]. Based on growth test or nitrogen balance assay, AA have been traditionally classified as nutritionally essential (indispensable) or non-essential (dispensable) for animals [1]. Animals have dietary requirements for both EAA (Met, Lys, Try, Thr, Phe, Ile, Val, Leu, and His) and NEAA to achieve maximum growth and production performance [34,35]. Some amino acids like arginine are considered to be semi-essential as they have to be provided in the diet to achieve optimal performance in the growth phase. According to the ideal model of FAO/WHO, a good balance of AA has to be provided for growing animals. Ratios of W_EAA_/W_TAA_ in the present study were all above 40%, and ratios of W_EAA_/W_NEAA_ were more than 60%. Furthermore, pig muscle AA concentrations in the present study were increased gradually with increasing age, suggesting, as expected an enhanced deposition of muscle protein along the pig development.

Meat flavor mainly depends on two aspects: taste and aroma [36,37]. The taste comes from flavor substances such as AA and small peptides, and aroma mainly derives from volatile substances released during meat cooking. Each AA contributes, to different degrees, to the taste of meat [38]. The active compounds in meat not only benefit the tastes as themselves, but also further react with each other, generating some aroma components through several pathways, such as the Maillard reaction [39]. The Maillard reaction between sulfur AA and reducing sugar can produce a meaty aroma [40]. An important observation in the present study is that Bama mini-pigs had higher muscle concentrations of Met, Phe, Tyr, Pro, and Ser in the nursery phase, and of Gly, Ala, and Ser in the growing phase than those of Landrace pigs. According to the above-mentioned rationale, the muscles of Bama mini-pigs can generate more taste-active compounds during the cooking process; a fact that is likely related to its quality in terms of organoleptic characteristics.

Dietary protein had minimal effect on muscle AA concentration in the current study. However, as the age increased, the AA pool was gradually regulated not only according to the dietary protein intake, but also according to the muscle type. In the LDM (type II fiber), pig genotype interacted markedly in the finishing phase with dietary protein intake regarding TAA, NEAA, and FAA. In addition, concentrations of the above-mentioned AA in Landrace pigs fed the higher protein-NRC diet were higher than those fed the GB diet. Concentrations of the above-mentioned AA in Bama mini-pigs fed the lower protein-GB diet were higher than those fed the NRC diet. In the BFM (type II fiber), pig genotype remarkably affected the AA pools in the nursery phase; whereas in the finishing phase, diet had a greater effect on most AA measured. Overall, the NRC diet increased concentrations of most AA more than the GB diet. On the other hand, our data suggest that the AA released from protein was highly muscle-dependent. The muscle enzymes, including calpains and cathepsins, are able to degrade myofibrillar proteins and release small peptides and AA. The different enzyme activities are responsible for the differences in the release of AA among the examined muscles [41]. The AA profiles in the present study may be related, at least in part, to the differences in muscle enzyme activity between LDM and BFM, but further experiments outside the scope of this study are required to test this hypothesis.

Skeletal muscle cells were the first cell type shown to arise through the activity of a single DNA binding transcription factor. This factor, named myogenic determining factor (MyoD), represented a paradigm for cell specification [42,43], and its discovery triggered an increasing interest for the identification of acting transcription factors such as myogenic regulatory factors (MRFs) [44], myocyte-specific enhancer binding factor 2 (MEF2) [45,46], and transforming growth factor beta (TGF-β) [47,48]. Previous studies have indicated that MRFs, including MyoD, MyoG, myogenic factor 5 (Myf5), and myogenic factor 6 (Myf6), function in processing myogenesis, and their expression has been used as an indicator of muscle development [49] and meat quality [50]. In each stage of myogenesis, different MRFs show various functions. In our study, expressions of MyoD, MyoG, and MEF2A in LDM, and MEF2A in BFM increased as the age increased, in association with enhanced muscle growth. In addition, the regulatory effect of MRF on the generation of skeletal muscle also depends on the interaction between cells and other related factors, such as MEF2 family factor interactions [51]. This factor is also involved in the regulation of skeletal muscle growth, by controlling the muscle cell differentiation and proliferation [52]. In the present study, the mRNA levels for MyoD, MyoG, and MEF2A in LDM of Bama mini-pigs were higher than in Landrace pigs, in accordance with the previous study by Wang et al. [53]. These latter authors reported that the expression levels of MyoD and MyoG in the Lantang pigs, another indigenous Chinese pig genotype, were higher than in Landrace pigs. Interestingly, the mRNA levels for MSTN in LDM and BFM of Bama mini-pigs were higher than in Landrace pigs. MSTN, belonging to the superfamily of TGF-β, is a glycoprotein widely expressed with functional specificity in skeletal muscle [54]. Indeed, high expression of MSTN in transgenic animal resulted in muscle atrophy [55], and the mutation of MSTN in cattle caused the so-called "double" muscle phenotype [56,57]. Early studies suggested that MEF2 may regulate the muscle fiber type conversion by combining with related sites of MSTN promoter [58]. Based on this notion, although Bama mini-pigs had increased mRNA levels for MyoD, MyoG, and MEF2A, the MSTN expression was however higher than in Landrace pigs. This may explain differences in the muscle growth between Landrace and Bama mini-pigs.

The phosphorylation of p70S6K and 4EBP1 by mTOR and the phosphorylation downstream of ribosomal protein S6 and EIF-4B stimulate translational initiation and contribute to cell growth. Protein deposition is linked with animal growth periods. In the present study, the expression levels of most proteins of the mTOR signaling pathway decreased with increasing age, including 4EBP1 and p-4EBP1 in LDM, and AKT, p-AKT, mTOR, p-mTOR, 4EBP1, p70S6K, p-p70S6K, and ratios of p-mTOR/mTOR and p-AKT/AKT in BFM of pigs, regardless of pig genotype or dietary protein level. Our data are consistent with a previous study by Kimball et al. (2002) which reported that the fractional rate of protein synthesis in pig skeletal muscle is high at birth and declines with age; meanwhile, the muscle abundance of mTOR decreased by 75% in 26- *vs*. 7-d-old pigs [59].

Using various technologies of two-dimensional electrophoresis, proteomic and transcriptomic analysis, researchers found that the skeletal muscle protein expression profiles were greatly different among pig genotypes [60–62]. In this study, pig genotype had a significant effect on the skeletal muscle protein expression profiles. The protein abundances of p-AKT, mTOR, p-mTOR, and p70S6K, and ratios of p-mTOR/mTOR, p-AKT/AKT, and p-p70S6K/p70S6K in LDM, and of AKT and p-AKT in BFM were higher in Landrace pigs than in Bama mini-pigs. These results indicate that genotype differences in growth performance and meat quality might be related to various expression levels of protein deposition- and muscle growth-related proteins.

Nutrient-mediated increases in muscle protein synthesis preserve the net muscle protein equilibrium (fasted losses *vs*. fed gains), which ensures a constant muscle mass at a given developmental stage. Nutritional factors, such as Gln [63,64], glutamate [65], putrescine [66], and amino acid mixture [67], have direct and/or indirect effects on the mTOR pathway or on the effectors of the mTOR pathway. S6K1 and 4EBP1, two downstream target proteins of mTOR pathway [68], are involved in the regulation of synthesis and metabolism, including synthesis of protein and ribosome, and biosynthesis and catabolism of mitochondria [69]. A previous study found that AA starvation could lead to dephosphorylation of S6K1 and 4EBP1 in cultured mammalian cells [70]. In skeletal muscle, physiological levels of AA stimulate the phosphorylation of mTOR, which, in turn, enhances the phosphorylation of S6K1 and 4EBP1, and leads to an increased synthesis of proteins. Interestingly, the dietary protein level in this study had a significant effect on the mTOR signaling pathway with interaction according to the pig genotype studied. The protein abundances of mTOR and p70S6K in Bama mini-pigs fed the lower protein-GB diet were higher than those fed the NRC diet. In contrast, the protein abundance of p70S6K and ratio of p-mTOR/mTOR in Landrace pigs fed the higher protein-NRC diet were higher than those fed the GB diet. These results suggest that high protein intake may not always be positively related to the rate of muscle protein synthesis and increase in the skeletal muscle mass, challenging the results of several previous studies [71,72]. In Bama mini-pigs, the low-protein diet enhanced the protein translation, which was beneficial regarding the AA pool and protein synthesis. These findings suggest that the utilization of nutrients in Landrace pigs and Bama mini-pigs are different.

In conclusion, our study indicates interactions between genotype and age for most AA regarding their concentration in skeletal muscles and protein synthesis-related signaling pathways. The concentrations of several AA related to taste and aroma at the early stage in Bama mini-pigs, and mRNA levels for MyoD, MyoG, MEF2A, and MSTN were higher than in Landrace pigs, while protein and phosphorylated levels of AKT, mTOR and p70S6K, and ratios of p-mTOR/mTOR, p-AKT/AKT, and p-p70S6K/p70S6K were lower. In addition, the lower protein-GB diet increased the protein abundances of mTOR and p70S6K in Bama mini-pigs, but decreased the protein abundance of p70S6K in Landrace pigs. The higher protein-NRC diet increased ratio of p-mTOR/mTOR in Landrace pigs. These findings suggest that variations in AA deposition and protein synthesis are greatly regulated by dietary protein level, being different according to pig genotype, developmental stage, and muscle type. Our study not only provides an important basis for further studies aiming at deciphering the molecular mechanisms responsible for differences in growth rate and meat quality between different pig genotypes, but also contributes to the optimization of animal feeding.
